# “*The power imbalance was blown out the window*”: developing and implementing creative workshops to enhance communication of statistics in patient and public involvement in clinical trials

**DOI:** 10.1186/s40900-024-00560-8

**Published:** 2024-03-20

**Authors:** Beatriz Goulao, Susan Morisson

**Affiliations:** 1 Health Services Research Unit, University of Aberdeen, Aberdeen, Scotland; 2 Creative professional, Edinburgh, Scotland

## Abstract

**Background:**

Despite the importance of statistical and numerical aspects in key decisions related to clinical trials and their impact in patient’s care, patient and public involvement remains underdeveloped in this field. Communication is a barrier to enable successful involvement of patients and the public in numerical aspects. Treatment important differences, a crucial numerical aspect in trials, is considered a priority for patient and public involvement. Creative methods have been proposed to improve communication of technical concepts with members of the public; and to democratise and improve inclusivity in patient and public involvement in health research.

**Methods:**

Working with creative professionals, public partners, and statisticians, we aimed to develop, pilot and implement creative workshops to promote a shared understanding of treatment important differences; and co-develop creative prototypes that could be used to communicate the statistical concept to a wider audience in the future. Three 2 to 4 h creative workshops based in the UK were delivered. The first two workshops included 22 participants. They were online and worked as pilots to refine the final in-person workshop via participant feedback and discussion. The final workshop focused on treatment important differences, and we collected information from participants on expectations, subjective numeracy, and experience.

**Results:**

The final workshop included 13 participants (5 creative professionals, 4 public partners, and 4 clinical trial statisticians). Participants reported creative workshops helped improve communication of treatment important differences between stakeholders reaching a common understanding of their meaning; and helped democratise knowledge exchange. Each group developed a creative prototype to communicate about treatment important differences with a wider audience, including a song, game, and a cartoon. Participants recommended the format to improve communication of other statistical or complex concepts between stakeholders.

**Conclusions:**

Creative workshops can promote shared understanding of complex, statistical concepts and co-development of creative outputs amongst stakeholders. Future work should explore generalisability of the intervention, and what outcomes might be important to consider when implementing creative workshops in patient and public involvement practice.

**Supplementary Information:**

The online version contains supplementary material available at 10.1186/s40900-024-00560-8.

## Abstract (plain language summary)

Patient and public involvement in clinical trials helps improve their relevance. Decisions related to numbers in trials consider information related to patients and their clinical experience, but patients and the public are rarely involved in these decisions. One barrier to achieve this is communication: numbers can be challenging to discuss. Creative methods (including writing, dancing, drawing) have been suggested as a potential way to improve communication of numbers with a wide audience. Working with creative professionals, public partners, and statisticians we developed, piloted, and implemented a creative workshop focusing on improving communication by reaching a common understanding between participants of a specific numerical concept related to clinical trials and where patient and public involvement is crucial. The creative workshop aimed to facilitate mutual learning between creative professionals, members of the public, and statisticians; and to promote co-development of creative outputs to describe the same numerical concept to a wider audience. Workshop participants felt the creative workshops improved communication of the numerical concept and helped everyone feel heard. Workshop participants were particularly interested in visual methods to support communication, and recommended creative workshops should be used to improve communication of other statistical and complex concepts.

**Supplementary Information:**

The online version contains supplementary material available at 10.1186/s40900-024-00560-8.

## Background

Patient and public involvement is essential to ensure the relevance and impact of health research [1]. Numerical and statistical aspects underpin quantitative health research: from the specific research question the research aims to address to the interpretation and communication of findings. Despite the importance of numerical and statistical aspects, patient and public involvement has often been neglected in this area [2–5]. The potential for patient and public involvement to increase the relevance of statistics and numerical aspects of research has been previously highlighted [3, 6–8]. In addition, our previous research showed an interest from public partners to be involved in discussions about numerical aspects of research [6, 7] and a willingness from researchers to make this happen [2, 9], but challenges in communication of statistical concepts and data were a key barrier [2, 10, 11].

Creative methods take an arts-based approach, where different types of arts—for example the visual arts, performing arts or the use of games and immersive installation—are used to reach a pre-established goal (i.e. enhance data literacy or improve the public’s understanding and involvement in research in an accessible way) [12].

Creative methods have been proposed in patient and public involvement activities [12–15] including to represent illness experiences through creative outputs [13] or to enhance communication of data terminology [16] and statistical methodology research [11]. Patient and public involvement literature suggests that creative approaches have the potential to reach new and diverse audiences [17]; facilitate co-creation of knowledge [15]; and build sustainable partnerships [12]. Artistic methods in patient and public involvement are often implemented for 2 main reasons [17]: 1. The arts offer a space where public partners in research are more willing to engage; 2. Artistic methods offer a potential to capture thoughts and ideas that are expressive, emergent and to an extent democratic.

Clinical trials produce the best evidence to decide which treatments should be available in healthcare and, therefore, can have a major impact in patients’ lives. Treatment important differences (which are sometimes called target differences) are the number one priority for patient and public involvement in numerical aspects of clinical trials according to a priority setting involving public partners and researchers [6]. They represent a difference between two treatments that make stakeholders (including patients) select one treatment over another. They are crucial in the interpretation of clinical trial results [18]. Even though different types of knowledge are key to determine appropriate treatment important differences (e.g. statistical, clinical and experience-based knowledge), patient and public partners are rarely directly involved in determining them [5, 19, 20]. A key barrier raised to improving involvement in treatment important differences (and in statistical and data aspects of research, in general) is communication and the common use of jargon in meetings [2]. Creative methods have been proposed to improve communication of technical concepts [16], and enhance data literacy for a general audience [21]. For this reason, we aimed to develop and refine creative workshops using arts-based approaches to facilitate patient and public involvement in treatment important differences in clinical trials. Our objectives were: (1) to develop shared understanding between creative professionals, public partners, and statisticians of a specific statistical concept—treatment important differences – and (2) to facilitate the co-development of creative prototypes to enable communication of this concept to a general audience in the future. We hypothesised that using creative workshops could enable democratic participation in discussions allowing all stakeholders (from the technical to the non-technical) to express their ideas and promoting a deliberative knowledge space which, in turn, can lead to more meaningful patient and public involvement in methodological aspects of research [8]; and that creative workshops can engage a diverse audience from a numeracy point of view.

## Methods

### Developing and piloting the creative workshop approach

The creative approach we propose follows the rationale of the wider creative methods literature described previously regarding literacy, dialogue, and empowerment. The GRIPP2 (Guidance for Reporting Involvement of Patients and the Public) checklist [22] was followed and completed (see Additional file 1). Table 1 summarises the creative workshop approach development from two online pilot workshops to the final workshop described in detail in the next section. We conceptualised the creative workshops as a way to support PPI in advance of making research project decisions allowing all involved to have a common understanding of the concepts discussed in decision making.

**Table 1:** Summary of the different creative workshops

	Workshop 1	Workshop 2	Workshop 3
Phase	Pilot/development	Pilot/development	Final implementation
Setting	Online	Online	In-person
Aim	To discuss several statistical concepts and develop creative ideas to communicate them with the public; to test and refine the creative workshop approach	To discuss creative ideas generated in creative workshop 1, and how they resonated with public partners; to test and refine the creative workshop approach	To reach a shared understanding of treatment important differences between stakeholders; and develop specific creative outputs (or prototypes) to communicate the concept to a wider audience
Participants	15 participants including creative professionals, public partners, and statisticians	7 public partners	12 participants including creative professionals, public partners and statisticians
Evaluation	Online feedback collected about experience post-workshop	Online feedback collected about experience post-workshop	Online feedback collected about experience pre and post-workshop; in-person, group feedback focusing on reflective learning

The initial creative workshop structure was developed by BG (lead researcher) based on wider literature [16, 23] and discussed in detail with Susan Morrison (SM, lead creative professional). Two initial, pilot creative workshops were undertaken aiming to develop and refine the method. The first online workshop focused on building a shared understanding between creative professionals, public partners and statisticians of statistical concepts and use creative methods to describe those concepts; the second one invited a group of public partners to review the wording and creative ideas used to describe the statistical concepts and refine the wording and ideas to be used in a public facing blog (https://pointrials.blogspot.com/).

Recruitment of workshop participants happened via social media (e.g. Twitter), People in Research (https://www.peopleinresearch.org/), and using the lead researcher and lead creative professional’s networks. Creative professionals and public partners were compensated for their time. Participants were expected to be based in the UK to ensure a common patient and public involvement culture, but there was no screening or eligibility criteria beyond that. Participants were selected based on availability to participate in the available dates. We stopped recruitment once we had reached the number of participants we were looking for. The number of participants we aimed to recruit was based on feasibility of implementing the creative workshop and specifically the small group discussions and it was based on previous experiences with group deliberative approaches that included both researchers and public partners, specifically the James Lind Alliance approach [6].

Fifteen participants took part in workshop 1 (five creative professionals–including writers and performers, four public partners, six clinical trial statisticians). It consisted of a 2-h session with an initial introduction and creative icebreaker to the whole group, followed by breakout rooms to allow smaller groups discussion of specific statistical concepts. Each small group consisted of by least one creative professional, one public partner, and one statistician. Workshop 2 lasted 2 h and included seven public partners. Both workshops were facilitated by BG; SM co-facilitated workshop 1 and was also a participant.

In workshop 1, the introduction reiterated the workshop aims and allowed attendees to meet. Each smaller group was allocated a statistical concept (e.g. missing data in clinical trials) in advance and had time to discuss it and develop creative ideas on how to communicate it. The groups were given no specific limits on what the ideas might be (from analogies and stories to visualisations) but were told their ideas and suggestions would be used to communicate about that specific concept in a patient and public involvement setting (i.e. not clinical). The groups were brought back into a main virtual room to share learnings and ideas. In workshop 2, participants also started in the main room and did a creative icebreaker. They were then allocated to breakout rooms to discuss different statistical concepts and their communication.

Feedback for both workshops was collected via online surveys using Google Docs immediately after each workshop. Feedback collected was voluntary and anonymous. Participants were informed it would be used to inform future creative workshops and could be included in publications related to the exercise. The feedback form asked “Was the session what you expected? Whether the answer is yes or no, please tell us why”, “What did you enjoy the most about the session today?”, “How can we improve future sessions?”. Thirteen out of fifteen participants provided feedback to workshop 1; all seven participants in workshop 2 provided feedback. The feedback collected informed the in-person creative workshop method implemented to produce tools to communicate about treatment important differences.

Workshop participants were very positive about its format and aims (“*It was great fun, informative and a truly interesting exploration into the language of stats.”*; *“The session was amazing and I have to say it's the best I have been to. These were all challenging topics and I felt we did so well to come together and tackle them.”*) and the novel interaction with creative professionals *(“Having the group facilitation done by creatives brought a whole new sense of fun, reality and enjoyment to the proceedings.”*). The key strengths mentioned were the ability to learn from others and understand new ideas; the creative process; the creative icebreaker that set the mood; the democratisation of the process *(“The power imbalance was blown out the window”*). The key suggestions for improvement were: more time or fewer statistical concepts to allow in-depth discussions; knowing in advance what statistical items will be discussed; having a more clear definition of what creative prototypes entail; in-person workshops. Through the conduct of online workshops 1 and 2, it became clear that the 2-step approach was unnecessary and potentially detrimental as public partners in workshop 2 did not get a chance to discuss the creative ideas with statisticians or creative professionals.

## Implementing the final creative workshop

**Fig. 1:**
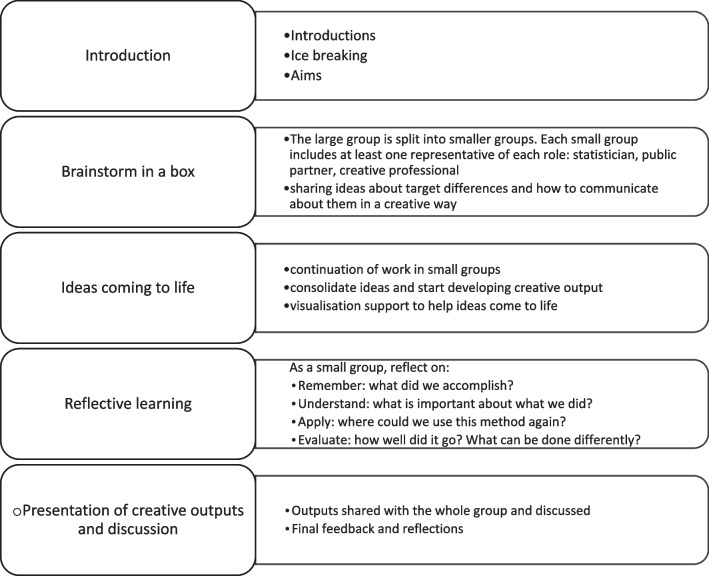
Content of final workshop

Following the feedback from the pilot creative workshop, BG (lead researcher) refined the creative workshop approach and discussed a new version in detail with SM (lead creative professional). The final creative workshop was held in-person in Nov 2022. Figure 1 presents its detailed structure. Based on priorities for patient and public involvement in numerical aspects of trials [6], we selected the number one priority focusing on a single statistical concept (treatment important differences) with two variations (clinically important differences [18], non-inferiority margins [20]) to focus the workshop. Information about treatment important differences including applied examples and further reading was sent to participants in advance of the workshop. Participants also received information about each other in advance of the workshop (i.e. short bios), and an agenda with specific aims.

Recruitment routes and processes, as well as compensation, replicated what happened in the pilot workshops.

Participants were asked to focus on the process of producing the creative outputs, and their learnings throughout, rather than the actual creative outputs developed.

**Fig. 2:**
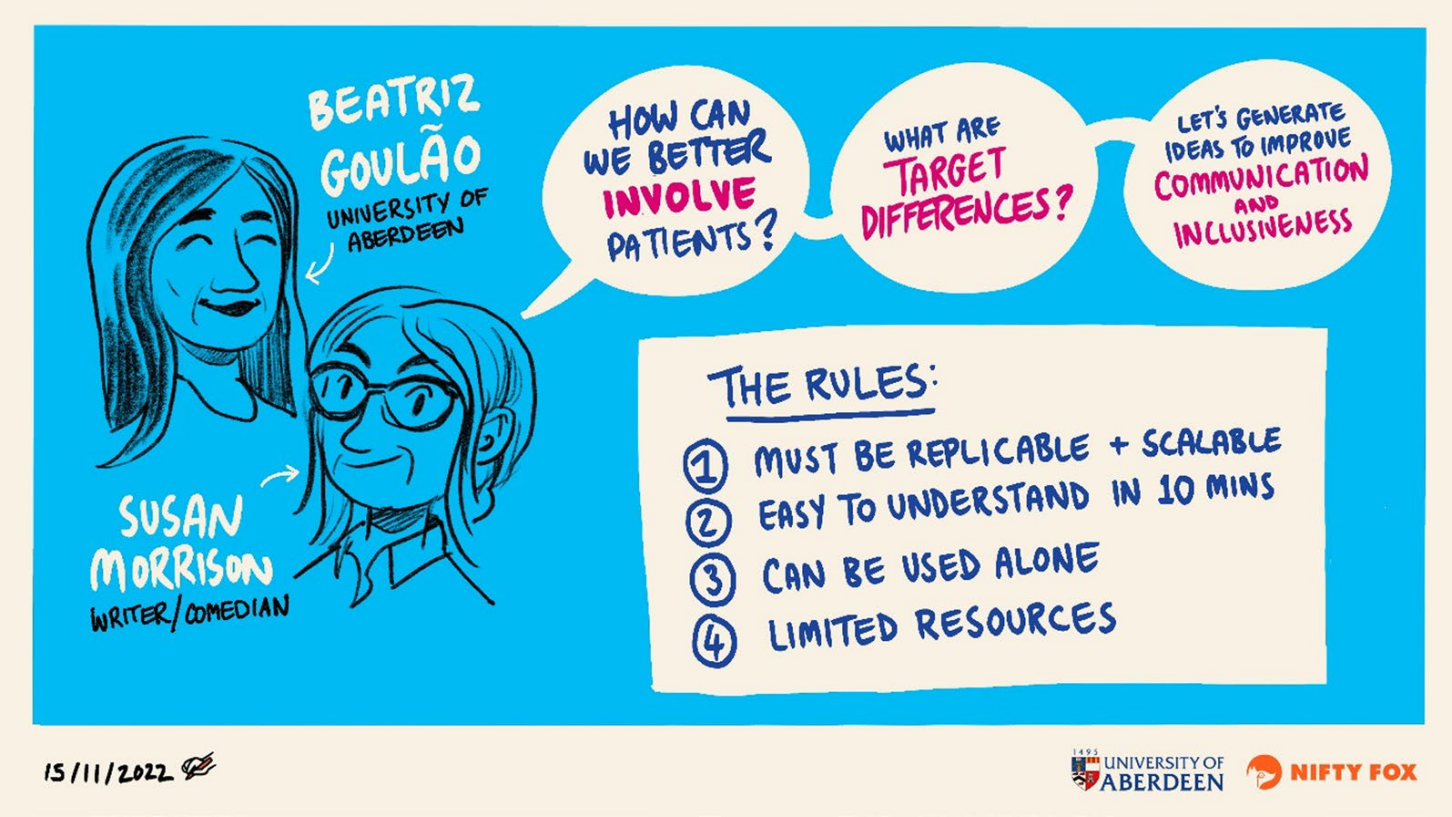
Final creative workshop aims and rules to develop prototype creative outputs

BG facilitated the workshop with the support of SM, who was also a participant. The workshop started with an introduction reviewing its aims, and it added ground rules for the brainstorming of creative prototypes including that the prototype must be: 1. replicable and scalable so it can reach a wider audience; 2. easy to understand and consumed in ten minutes at the most; 3. used by itself without any additional support required; 4. Possible to develop with limited resources (i.e. up to 10k of funding) (Fig. 2). The group was then split into smaller groups of up to four to discuss 2 types of treatment important differences: 2 groups discussed clinically important, and 2 groups discussed non-inferiority margins.

## Data collection and analysis

Before the workshop started, we collected data on expectations from attendees and their numeracy levels using the validated Subjective Numeracy Scale-3 [24] via an online Google Docs form. The Subjective Numeracy Scale (SNS)-3 consists of three questions, 2 focusing on self-reported numeracy skills and one on subject preference. The scale varies from 3 (lowest subjective numeracy) to 18 (highest). SNS-3 data is presented using descriptive statistics including a commonly used categorisation [25] of low (3–12), medium (13–15) and high subjective numeracy (16–18) for comparison purposes. At the end of the workshop, prototypes and experience information were collected in situ for each group through a paper form and, immediately after the session, individually via an online survey using Google Docs. Both forms were anonymous. Group feedback forms focused on reflective learning and included four domains: what did we accomplish? What is important about what we did? Where could we use this method again? And how well did it go? What should be done differently?. The individual feedback forms asked:

1. What role do you identify most with? (e.g. statistician, public partner, creative professional)
2. Was the session what you expected? Whether the answer is yes or no, please tell us why.
3. What did you enjoy the most about the session today?
4. How can we improve future sessions? How could we use this method again?

Feedback collected (individually or as a group) was read and summarised by BG.

No ethical approval was sought as this was a consultation and involvement activity. All participants were compensated for their travel, subsistence, and accommodation for the in-person workshop.

## Results

There were 13 workshop participants in total including 5 creative professionals (visual artists, writer, songwriters, performers), 4 public partners, and 4 clinical trial statisticians. Two public partners, one creative professional, and one statistician had taken part in one of the two pilot workshops.

## Expectations and numeracy

Twelve out of 13 participants provided information on expectations and subjective numeracy. Participants’ expectations were generally positive hoping to learn more about statistics or gain confidence in discussing them, to learn from others and their perspectives, and/or improve their communication to patients and the public. Subjective numeracy scale results varied between 5 and 17 with a mean of 13.8, standard deviation of 3; low subjective numeracy (3–12) was reported by one participant (8%), medium subjective numeracy (13–15) was reported by most participants (n = 8, 67%) and high subjective numeracy (16–18) was reported by 3 participants (25%).

## Creative workshops are an engaging way to discuss statistical concepts in lay terms

Participants pointed out the ability to be creative in a fun environment, working in groups that included all different perspectives (creative, statistical, public) as strengths of the workshop. They saw the workshop as an opportunity for mutual learning, and highlighted the co-production of the creative ideas (i.e. everyone felt equally involved). Public partners and statisticians highlighted working with creative professionals as a unique and fun opportunity that allowed to build bridges between disciplines and experiences.

## A 2-step approach to learning and creating

**Fig. 3:**
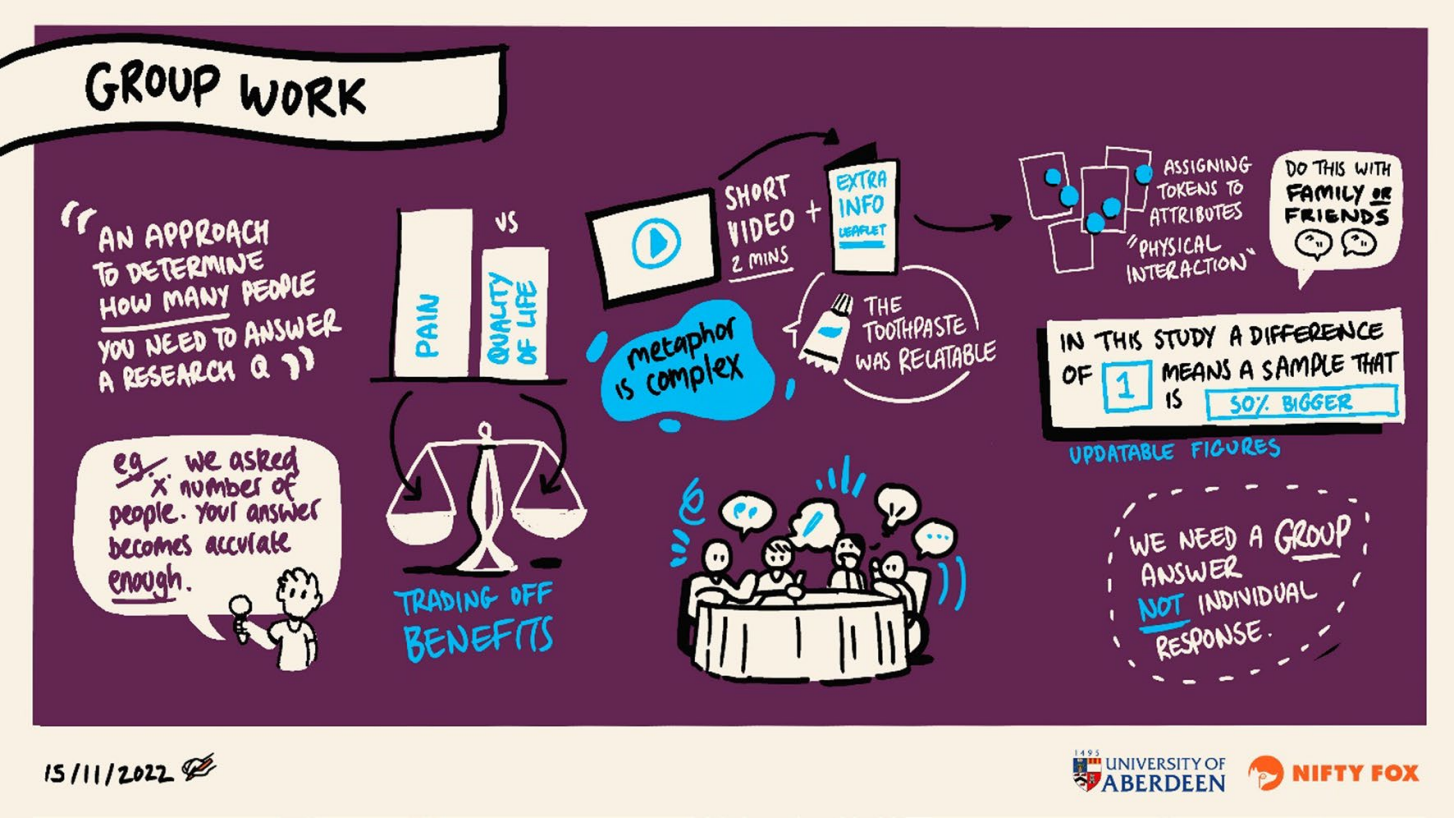
Illustration of the development of a shared understanding of target differences between participants in one small group

Participants’ descriptions of their experience improved understanding of the how the process worked for them. Participants felt the creative workshop approach worked in 2 steps: first, the smaller groups had to learn about the concept and how to communicate about it with each other, developing early ideas about stories and visualisations (mutual learning); second, the smaller groups had to develop a concrete prototype to illustrate their thoughts and discussions (creating). It was suggested that, in future workshops, these 2 steps should be clearer to participants from the start as well as explaining the rationale for the use of creativity to create prototypes to communicate statistical concepts. Figure 3 illustrates step 1’s initial discussions and learnings in one of the small groups.

## The importance of visualising

A key finding from the workshop was the importance of visualising the ideas developed. Participants suggested that resources should be readily available to visualise their ideas and that this led to a feeling of achievement of mutual understanding. Suggestions to improve future workshops included the addition of different tools to aid visualisation such as Lego, and other props.

## The prototypes

**Fig. 4:**
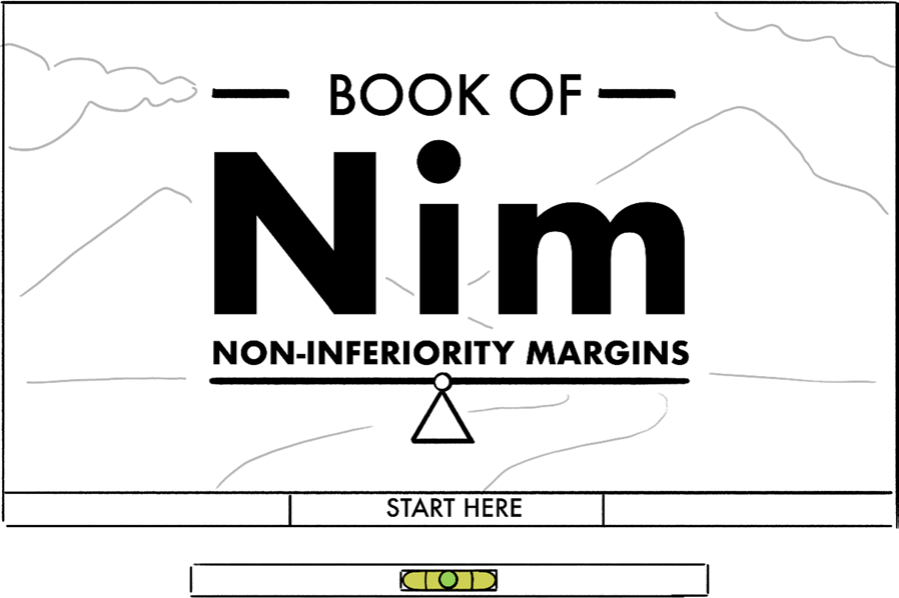
Example of a creative prototype developed at the creative workshop. This prototype involved an interactive book where players could select different realities that would take them to a series of decisions and trade-offs until they reached a final choice. Through the process players would learn about the concept of non-inferiority margins

Participants developed their prototypes during the workshop with or without support of a visual artist. At the end, each group presented their concept to the whole group. Examples of the creative prototypes presented included a book of non-inferiority margins (NIM) that would ask the player to make trade-offs until they reached their final decision on an acceptable margin (Fig. 4); a website with several layers increasing complexity of explanations about treatment important differences starting with a creative story to illustrate the concept in a simple way, but offering the opportunity to explore more technical literature if of interest. Other examples included a cartoon alien story with a song about treatment important differences.

## Future applications

There was consensus that the creative approach would help support communication and understanding of other statistical or data-related concepts. Participants also suggested this method could be easily adapted to discuss other complex topics (e.g. research methodology, medical jargon, treatment pathways, etc.) and with other target groups (e.g. children).

## Discussion

In this paper, we present the development, refinement, and implementation of creative workshops to improve communication about statistical concepts with public partners; and to co-produce creative prototypes that support wider communication of a statistical concept in patient and public involvement. This approach can enable meaningful patient and public involvement in treatment important differences by addressing a previously identified key barrier to patient and public involvement in statistics and data aspects of research: the need for better and clearer communication. The approach could be generalisable to other statistical, data or methodological research related concepts. Table 2 maps the approach to UK standards for patient and public involvement and engagement and highlights areas for improvement [13, 26].

**Table 2:** Creative workshops to enable PPI in statistics mapped onto UK standards for public involvement and engagement with considerations of areas for improvement

UK standard	Summary	Activity – how the standard was met	Areas for improvement
Inclusive opportunities	Offering public involvement opportunities that are accessible and reach people and groups according to research needs	The creative approach was developed to ensure it is inclusive of different numeracy levels	We need to put more effort in ensuring a range of numeracy levels when recruiting to future workshops; future work can expand on diversity of participants aiming to include people with different levels of general literacy, socioeconomic backgrounds, and ethnicities
Working together	Work together in a way that values all contributions, and that builds and sustains mutually respectful and productive relationships	All participants in the workshop – creative professionals, public partners, and statisticians – had equal say in the discussions and final prototype they produced. Participants worked collaboratively to achieve the aim of the workshop	This was highlighted as a key strength of this approach by participants
Support and learning	Offer and promote learning opportunities that build confidence and skills for public involvement in research	The workshop is by its nature a learning opportunity to enable PPI. It is a learning opportunity for all stakeholders involved – statisticians developed their communication skills, creative professionals learned about statistics and patient’s role in shaping them, and public partners learned about statistics and their impact in their lives	This was highlighted as a key strength of this approach by participants, and we included reflective learning in our assessment of the final creative workshop; however, there is scope to collect more information about learning outcomes and needs from all stakeholders that could enhance or expand future initiatives
Communication	Use plain language for well-timed and relevant communications as part of involvement plans and activities	The workshop’s aim was precisely to develop shared ways to explain a statistical concept in plain language; plain language was an essential feature to achieve the common goal of preparing a communication output as only the statistician in each group would know what a treatment important difference is from a technical perspective	This was highlighted as a key strength of this approach by participants; however, suggestions for improvement included clearer communication at the start of the creative workshop of a 2-stage approach to developing the final prototype, as well as presenting examples of possible prototypes
Impact	Seek improvement by identifying and sharing the difference that public involvement makes to research	Given the participatory nature of the workshop, it is challenging to pinpoint what the specific contribution of public involvement was; rather, this was a team effort, and therefore it is challenging to separate each stakeholder group contribution from the whole	More research needs to be done to assess the impact of the fully developed prototypes in the understanding and meaningful involvement of patients and the public in treatment important differences; and to explore different potentially important outcomes from taking part in creative workshops
Governance	Involve the public in research management, regulation, leadership and decision making	The activity developed here and its prototypes aim to enable patient and public partners to be involved in future decision making related to a specific statistical concept, treatment important differences	Implementing the creative workshop approach to enhance learning and develop prototypes to communicate about other statistical and data related concepts, or wider complex concepts can help facilitate PPI in decision making of a wide range of research. This is particularly promising in areas where a key barrier for PPI in decision making is communication such as methodological research

The creative workshop approach worked well in enabling a perception of shared understanding of treatment important differences and producing prototypes for creative outputs to support its communication to wider audiences. In line with previous literature using creative approaches to improve data literacy or to facilitate patient and public involvement [13, 14, 17], participants confirmed our hypothesis that creative methods can lead to a more democratic experience of discussions around statistical concepts. This is particularly important since tokenism remains a key barrier to meaningful patient and public involvement in health research [27]. Past initiatives to enhance data literacy via creative approaches have found focusing on the process is more important than focusing on the final output [28], and we support this observation. This allowed participants to prioritise reaching a shared understanding of treatment important differences before they focused on developing a creative prototype. Future workshops should incorporate participants’ suggestions including making the 2-step approach clearer and providing more examples of expected outputs; making visual artists or visual tools available to all small groups; and, aiming to have a final output ready to share with a wider audience, including post-workshop creative professional time compensation.

One of the key strengths of the creative workshop approach is its development building from previous empirical literature [16, 23] and working closely with an expert creative professional; as well as its refinement through the feedback and input of all participants including public partners. There is scope to further develop the theoretical underpinnings of the creative workshops [29] leading to better understanding of what creative methods might work better, or what outcomes are most meaningful to measure when evaluating creative workshops from different stakeholders’ perspectives. To ensure creative workshops can be replicable with different concepts, and in different contexts, a thorough evaluation of their process, and reflection on their “key ingredients” needs to be undertaken. This will ensure we better understand how the workshops work, to whom, and under what conditions. This recommendation is in line with the general art-based methods for public engagement with research literature: there is a need for more robust evaluation of processes and a bigger focus on the fidelity of arts-based methods to allow lessons to be learned beyond single projects [12].

The creative workshops have potential benefits for participants beyond building a shared understanding of topics and promoting a deliberative knowledge space; namely, they can increase data literacy for non-technical participants; and enhance communication skills and the understanding of the potential real-life impact of numbers and statistics for technical participants. However, we did not measure those impacts in this work, and propose these could be explored in future research. Even though we created multiple prototypes that could be developed into ready-to-use outputs to communicate about treatment important differences to a wider audience, we did not have the resources to compensate creative professionals to finalise their development or a specific plan for their dissemination. This is because our main focus was on the creative workshops’ process to develop a deliberative knowledge space and ensure mutual learning [8]. However, we aim to finalise the prototypes developed here, develop an appropriate dissemination plan to support their use with wider audiences, and measure their impact. This would mean the outputs can be reused to raise awareness about treatment important differences and their potential impact in treatments available to patients. Final workshop participants had a range of numeracy levels, and their average was in line with other general population groups [30], however we had a lower proportion of participants with low numeracy when compared with general research participants [31, 32]. Given art-based methods such as the creative workshops are expected to make research topics more accessible [12], future work should focus on recruiting a larger proportion of participants with low numeracy levels and explore the role and impact creative workshops can have on their involvement in numerical aspects of research. The need for additional resources, funding and researcher time may be a barrier to the future implementation of these workshops; however, these workshops can be key in enabling meaningful communication and involvement especially in complex concepts and, therefore, should be considered through a cost–benefit lens.

In conclusion, the creative workshop approach had positive impact on shared understanding of a statistical concept with potential to enhance data literacy skills for non-technical learners and communication skills for all involved including technical participants (i.e., statisticians). Importantly, this new and innovative approach has the potential to help overcome a key barrier in patient and public involvement in statistics in clinical trials or in data intensive research [33] by allowing clear, meaningful, and democratic communication about complex (statistical) concepts in research.

### Supplementary Information

**Additional file 1. Table 1** - GRIPP 2 short form.

## Data Availability

Not applicable.
