# Supplementary material for: “The power imbalance was blown out the window”: developing and implementing creative workshops to enhance communication of statistics in patient and public involvement in clinical trials
Source: Res Involv Engagem. 2024 Mar 20;10:32. doi: 10.1186/s40900-024-00560-8 (PMC10956209; doi:10.1186/s40900-024-00560-8)
Supplement: Supplementary file 1 — Additional file 1. Table 1 - GRIPP 2 short form. [file 40900_2024_560_MOESM1_ESM.docx]

Additional File 1: GRIPP 2 short form

| **Section and topic** | **Item** | **Reported on page No** |
| --- | --- | --- |
| 1: Aim | Report the aim of PPI in the study | 5 |
| 2: Methods | Provide a clear description of the methods used for PPI in the study | 5-9 |
| 3: Study results | Outcomes—Report the results of PPI in the study, including both positive and negative outcomes | 10,11 |
| 4: Discussion and conclusions | Outcomes—Comment on the extent to which PPI influenced the study overall. Describe positive and negative effects | 12, 13 |
| 5: Reflections/critical perspective | Comment critically on the study, reflecting on the things that went well and those that did not, so others can learn from this experience | Table 2, Pages 24-26 |
